# Supplementary material for: Automated craniofacial biometry with 3D T2w fetal MRI
Source: PLOS Digit Health. 2024 Dec 30;3(12):e0000663. doi: 10.1371/journal.pdig.0000663 (PMC11684610; doi:10.1371/journal.pdig.0000663)

S1 File: Supplementary Figures

Table of Contents

[Fig A. Image examples of SVR head image quality scoring for inclusion in the dataset, scores of 3 or 4 were considered adequate quality for inclusion in the study. 2](#_Toc180416333)

[Fig B. Boxplots of measurements stratified by healthy control (blue) and T21 groups (red). Central tendency is represented by the black horizontal line, and the upper and lower limits of the box represent the interquartile range (2nd to 3rd quartile). The black dots represent outliers 3](#_Toc180416334)

[Fig C. Human observer diagnostic confidence of label placement and measurement accuracy. a. Observer 0 ( repeated measures). b. Average of observers 1 to 3 (each performing single independent measurements 4](#_Toc180416335)

[Fig D. GA-matched fetal MRI datasets used in the control (104) and T21 (24) comparison a. GA distribution b. MRI protocols and fetal sex 5](#_Toc180416336)

[Fig E. Chart demostrating the proportion of biometrics that fell outside of normative range (5th - 95th percentile) for each T21 subject, upper chart: for most significant biometrics and lower chart all biometrics. 6](#_Toc180416337)

## **Fig A.** Image examples of SVR head image quality scoring for inclusion in the dataset, scores of 3 or 4 were considered adequate quality for inclusion in the study.


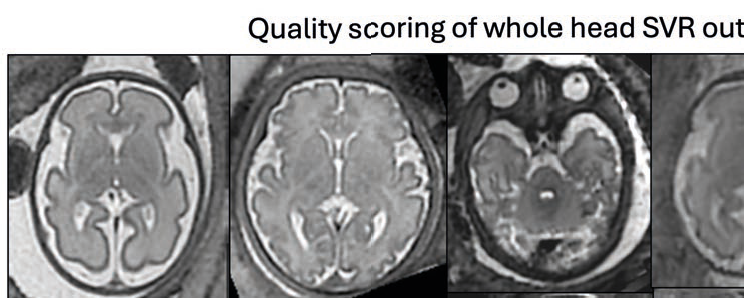

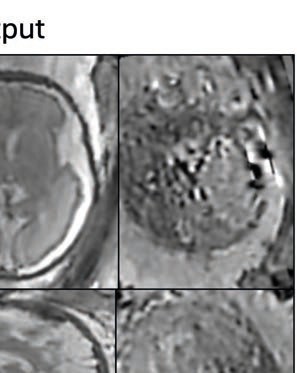

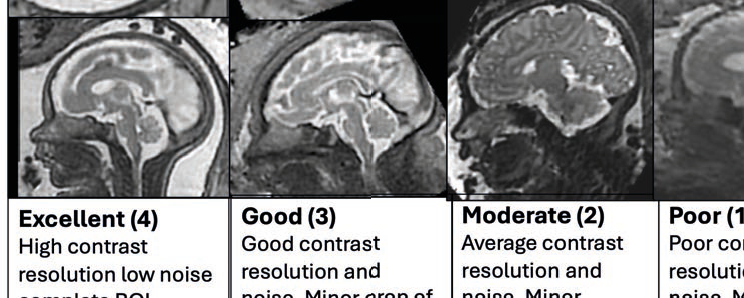

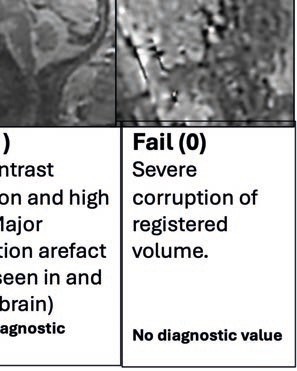

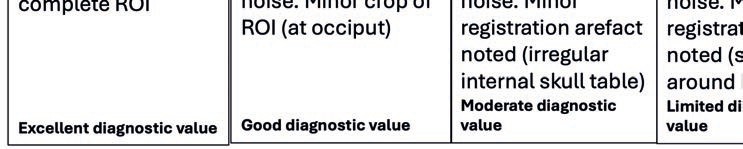


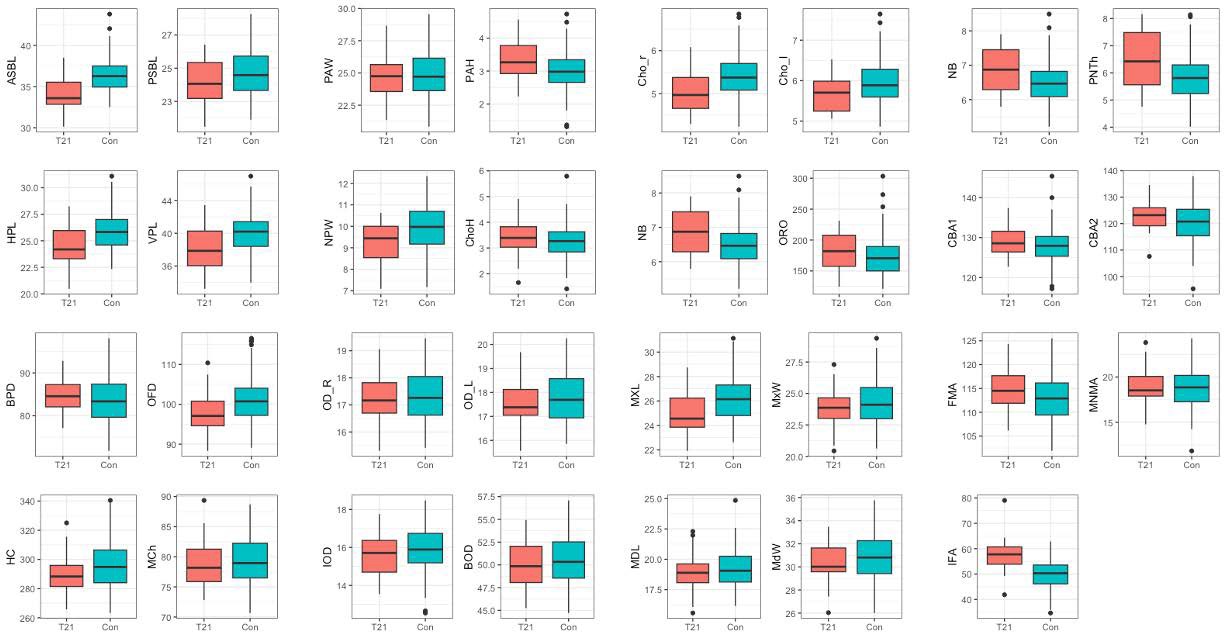


## **Fig B.** Boxplots of measurements stratified by healthy control (blue) and T21 groups (red). Central tendency is represented by the black horizontal line, and the upper and lower limits of the box represent the interquartile range (2nd to 3rd quartile). The black dots represent outliers

1. Confidence in 3D label placement (n=35) between first and second repeated observation


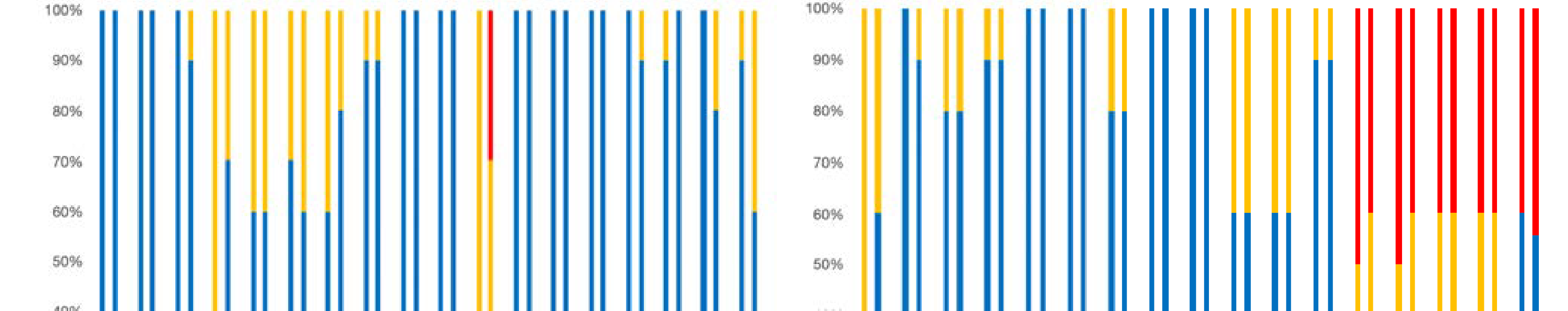

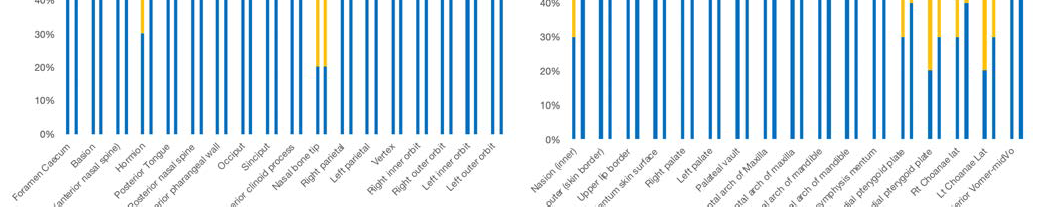

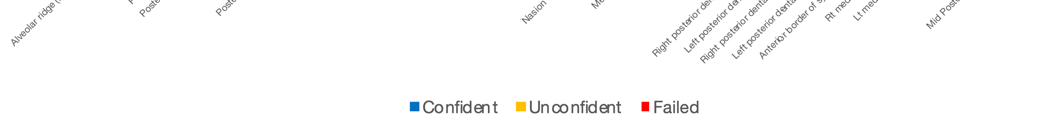


1.
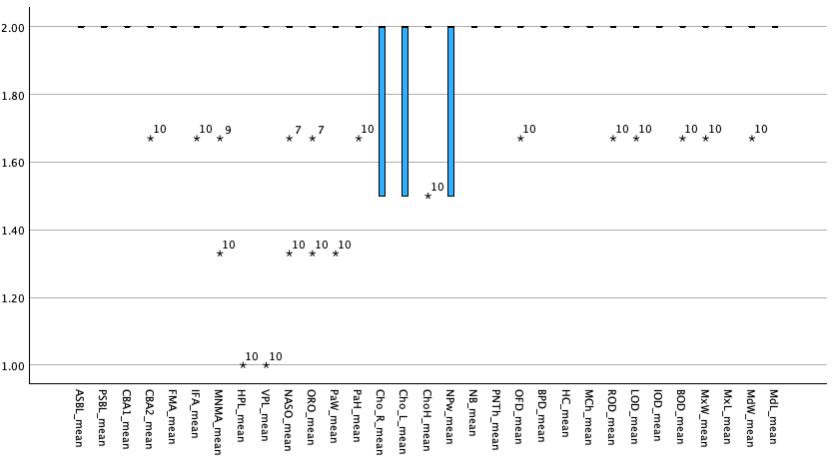
Boxplots of average confidence for 3 raters for obtaining measurements (scale: 0 = failed, 1 = unconfident, 2 = Confident). * Represents case number outliers, see S2 table 5. Note, small boxes at point 2 on the y-axis represents no variation.

## **Fig C.** Human observer diagnostic confidence of label placement and measurement accuracy. a. Observer 0 ( repeated measures). b. Average of observers 1 to 3 (each performing single independent measurements

## **Fig D.** GA-matched fetal MRI datasets used in the control (104) and T21 (24) comparison a. GA distribution b. MRI protocols and fetal sex


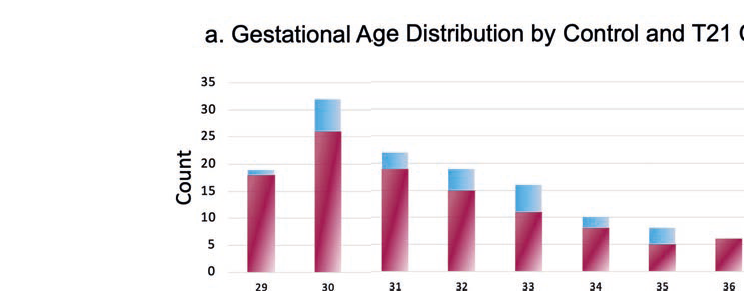

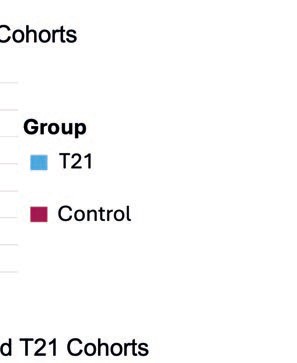

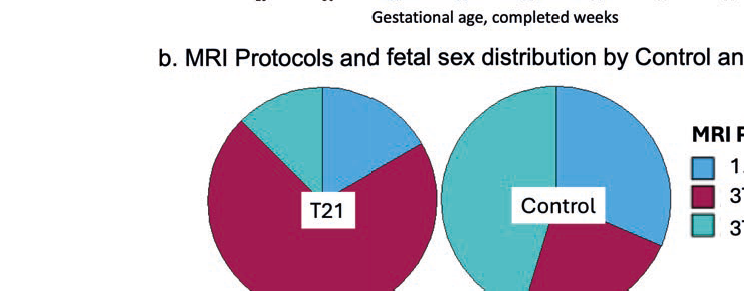

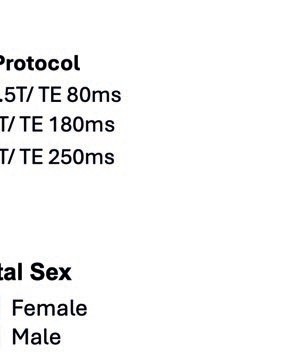

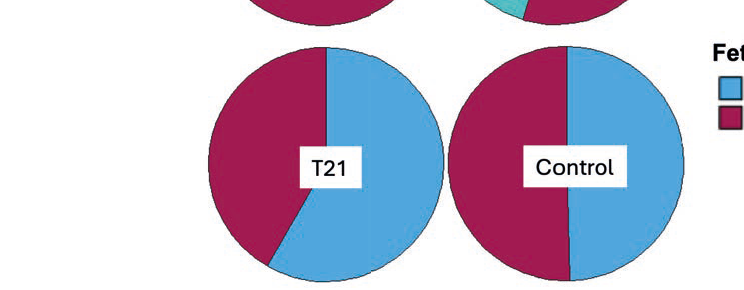

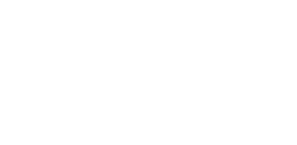


## **Fig E.** Chart demostrating the proportion of biometrics that fell outside of normative range (5th - 95th percentile) for each T21 subject, upper chart: for most significant biometrics and lower chart all biometrics.


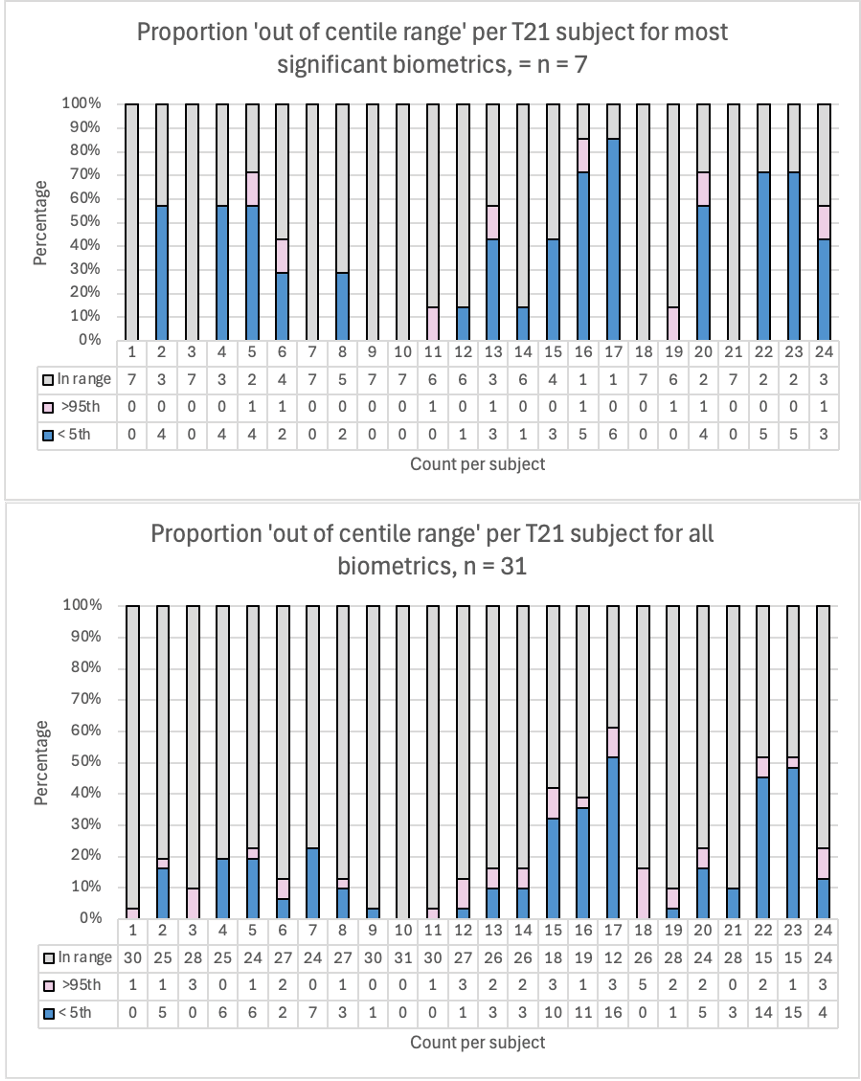

Supplement: S1 File — Fig B: Boxplots of measurements stratified by healthy control (blue) and T21 groups (red). Central tendency is represented by the black horizontal line, and the upper and lower limits of the box represent the interquartile range (2nd to 3rd quartile). The black dots represent outliers. Fig C: Human observer diagnostic confidence of label placement and measurement accuracy. a. Observer 0 (repeated measures). b. Average of observers 1 to 3 (each performing single independent measurements. Fig D: GA-matched fetal MRI datasets used in the control (104) and T21 (24) comparison a. GA distribution b. MRI protocols and fetal sex. Fig E: Chart demonstrating the proportion of biometrics that fell outside of normative range (5th - 95th percentile) for each T21 subject, upper chart: for most significant biometrics and lower chart all biometrics. (DOCX) [file pdig.0000663.s001.docx]
