# Supplementary material for: Automated craniofacial biometry with 3D T2w fetal MRI
Source: PLOS Digit Health. 2024 Dec 30;3(12):e0000663. doi: 10.1371/journal.pdig.0000663 (PMC11684610; doi:10.1371/journal.pdig.0000663)
Supplement: S2 File — Table B: Formalised measurement definitions for label-based craniofacial biometry protocol with 3D T2w fetal MRI. Table C: Formulae for automated biometry calculation based on 3D labels. Table D: Demographics of the 10 cases selected for quantitative evaluation of the proposed biometry protocol and pipeline. Table E: Results of absolute (upper table) and relative (lower table) differences with observer 0 (manual label based indirect measurement), observer 2 and 3 (direct manual measurement) and automated results compared to a single expert radiologist (direct manual measurement. Green represents relative mean difference or <10% or relative limits of agreement (+/− mean) of <20%. Orange represents automated measurements with more variation than any one manual observer. Table F: Table of Intraclass Correlation Coefficient (ICC) results. Green is and excellent or good agreement, yellow a moderate agreement, red is poor agreement (n.b. ICC threshold criteria interpreted only if Cronbach’s Alpha is >0.70). Table G: Summary of included datasets for the 3rd trimester T21 and age-matched control cohorts, by GA, MRI protocol, fetal sex and ethnicity. Table H: Table of number of subjects (n), mean measurement, and standard deviation (SD)—stratified by healthy control and T21 groups. Table I: Best linefit and Standard Deviation (SD) regression equations and method, for each biometric growth chart. GA = GA. Note: all SD regression formulae have linear bestfit method. Table J: ANOVA results for statistically significant biometry with largest effect size in T21 cohort (ABSL, HPL, VPL, OFD, IFA, NASO) and BPD as a non-significant results. Results corrected with robust standard errors. (DOCX) [file pdig.0000663.s002.docx]

S2 File: Supplementary Tables

Table of Contents

[Table A**.** 3D Anatomical Landmarks (labels) (*Landmark not used for biometry extraction) 2](#_Toc180416054)

[Table B**.** Formalised measurement definitions for label-based craniofacial biometry protocol with 3D T2w fetal MRI. 5](#_Toc180416055)

[Table C**.** Formulae for automated biometry calculation based on 3D labels 6](#_Toc180416056)

[Table D. Demographics of the 10 cases selected for quantitative evaluation of the proposed biometry protocol and pipeline. 7](#_Toc180416057)

[Table E. Results of absolute (upper table) and relative (lower table) differences 8](#_Toc180416058)

[Table F. Table of Intraclass Correlation Coefficient (ICC) results. 9](#_Toc180416059)

[Table G. Table of number of subjects (n), mean measurement, and standard deviation (SD) - stratified by healthy control and T21 groups 10](#_Toc180416060)

[Table H. Summary of included datasets for the 3rd trimester T21 and age-matched control cohorts, by GA, MRI protocol, fetal sex and ethnicity 11](#_Toc180416061)

[Table I. Best linefit and Standard Deviation (SD) regression equations and method, for each biometric growth chart. GA = GA. Note: all SD regression formulae have linear bestfit method. 12](#_Toc180416062)

[Table J. ANOVA results for statistically significant biometry with largest effect size in T21 cohort (ABSL, HPL, VPL, OFD, IFA, NASO) and BPD as a non-significant results 13](#_Toc180416063)

### Table A**.** 3D Anatomical Landmarks (labels) (*Landmark not used for biometry extraction)

| **Label number** | **Anatomical point** | **Abbr.** | **Description** |
| --- | --- | --- | --- |
| 1 | Foramen Caecum | Fc | The midline point marking the pit between the fetal crista |
|  |  |  | galli and the endocranial wall of the frontal bone |
| 2 | Basion | Ba | The midline point on the anterior margin of the foramen |
|  |  |  | magnum |
| 3 | Alveolar ridge (anterior nasal spine) | ANS | The midline central point on the tip of the alveolar ridge |
|  |  |  | (also synonymous with anterior nasal spine, ANS) |
| 4 | Hormion | H | The posterior-most midline point on the junction between |
|  |  |  | the ventral surface of the sphenoid and the vomeral root |
|  |  |  | (also, point located at the intersection between the perpen- |
|  |  |  | dicular line to S-Ba from PNS and the cranial base) |
| 5 | Posterior Tongue | TP | Posterior most aspect of the tongue in the midline |
| 6 | Posterior nasal spine | PNS | The midline point of the superior surface of the hard palate |
|  |  |  | where the horizontal aspect of the palatine bone meets the |
|  |  |  | posterior most aspect of the vomer bone. |
| 7 | Posterior pharangeal wall | PPW | The posterior point of the pharanyx on a linear line con- |
|  |  |  | tiguous with the hard palate length (ANS-PNS) |
| 8 | Occiput | Oc | Posterior most skull border in axial plane at level of tha- |
|  |  |  | lami |
| 9* | Cervical axis | C1 | The midline point on the first vertebral body anterior to |
|  |  |  | the basion point |
| 10 | Sinciput | Si | Anterior most skull border in axial plane at level of thalami |
| 11 | Posterior clinoid process | PCP | The midline point on the posteior raised tuberculum sella |
|  |  |  | of the body of the sphenoid |
| 12 | Nasal bone tip | NB | Inferior most tip of the nasal bone |
| 13* | Nose tip | NBT | Tip of the superficial surface of nasal soft tissue |
| 14* | Tongue root | TR | Most inferior posterior muscular part of the tongue (esti- |
|  |  |  | mated site of hyoid bone) |
| 15 | Right parietal | Pt_r | Right lateral most skull border in axial plane at level of |
|  |  |  | SI-Oc plane (OFD/BPD) |
| 16 | Left parietal | Pt_l | Left lateral most skull border in axial plane at level of |
|  |  |  | Si-Oc plane (OFD/BPD) |
| 17 | Vertex | Ve | Most superior skull border in a midline sagittal plane |
|  |  |  | (at level of cerebral peduncles when oriented to brain |
|  |  |  | orthgonal planes in coronal) |
| 18 | Right palate | pa_r | Right mid-inferior border of posterior most maxillary mo- |
|  |  |  | lar in coronal plane (when oriented for coronal facial |
|  |  |  | views) |

Continued on next page

Table A – Continued from previous page

| **Label number** | **Anatomical point** | **Abbr.** | **Description** |
| --- | --- | --- | --- |
| 19 | Left palate | pa_l | Left mid-inferior border of posterior most maxillary molar |
|  |  |  | in coronal plane (when oriented for coronal facial views) |
| 20 | Palateal vault | PaV | Midline point in coronal plane at level of posterior most |
|  |  |  | maxillary molar at the inferior border of the hard palate |
| 21 | Right posterior dental arch of Max- | Mx_r | Posterior point of last right molar tooth socket at the level |
|  | illa |  | of the widest point of the maxillary dental arch |
| 22 | Left posterior dental arch of maxilla | Mx_l | Posterior point of last left molar tooth socket at the level |
|  |  |  | of the widest point of the maxillary dental arch |
| 23 | Right posterior dental arch of mandible | man_r | Posterior point of last right molar tooth socket at the level of the widest point of the mandibular dental arch (at the |
|  |  |  | level of the masseter on its entrance point to the mandible) |
| 24 | Left posterior dental arch of mandible | man_l | Posterior point of last left molar tooth socket at the level of the widest point of the mandibular dental arch (at the |
|  |  |  | level of the masseter on its entrance point to the mandible) |
| 25 | Anterior border of symphysis men- | Me | Midline point at the level of the symphysis mentis of the |
|  | tum |  | mandible |
| 26* | Right Superior external ear | hel_r | Right most superior point of the helix (external ear) |
| 27* | Right inferior external ear | lob_r | Right most interior point of the auricular lobule (external |
|  |  |  | ear) |
| 28* | Right external auditory meatus | EAM_r | Midpoint of the right external auditory meatus (between |
|  |  |  | the tragus and the concha) |
| 29* | Left Superior external ear | hel_l | Left most superior point of the helix (external ear) |
| 30* | Left inferior external ear | lob-l | Left most interior point of the auricular lobule (external |
|  |  |  | ear) |
| 31* | Left external auditory meatus | EAM_l | Midpoint of the left external auditory meatus (between the |
|  |  |  | tragus and the concha) |
| 32 | Right inner orbit | RO_in | Right inner border of the orbital globe (i.e. medial outer |
|  |  |  | edge of sclera) at widest point in axial view |
| 33 | Right outer orbit | RO_o | Right outer border of the orbital globe (i.e. lateral outer |
|  |  |  | edge of sclera) at widest point in axial view |
| 34 | Left inner orbit | LO_in | Left inner border of the orbital globe (i.e. medial outer |
|  |  |  | edge of sclera) at widest point in axial view |
| 35 | Left outer orbit | LO_o | Left outer border of the orbital globe (i.e. lateral outer |
|  |  |  | edge of sclera) at widest point in axial view |
| 36 | Nasion (inner) | NaIn | Inner border of nasion |
| 37 | Nasion outer (skin border) | NaO | Outer skin border of nasion |
| 38* | Nuchal fold inner | NF_in | Nuchal inner border (medial subcutaneous layer) |
| 39* | Nuchal fold outer (skin border) | NF_o | Nuchal outer border (posterior border subcutaneous layer) |
| 40 | Upper lip border | Lip | Skin border of the upper lip midline |

Continued on next page

Table A – Continued from previous page

| **Label number** | **Anatomical point** | **Abbr.** | **Description** |
| --- | --- | --- | --- |
| 41 | Mentum skin surface | Chin | Skin border of the chin midline |
| 42* | opisthion | Op | Midline posterior border of the foramen magnum |
| 43* | Right Lateral Foramen Magnum | FM_r | Right lateral border of the foramen magnum |
| 44* | Left lateral Foramen Magnum | FM_l | Left lateral inner border of the foramen Magnum |
| 45* | Tongue tip | TT | Most anterior part of tongue in the midline |
| 46 | Rt medial pterygoid plate | MPP_r | Point on the right posterior lateral border of the nasal |
|  |  |  | labrinth at a level of the vomer |
| 47 | Lt medial pterygoid plate | MPP_l | Point on the left posterior lateral border of the nasal |
|  |  |  | labrinth at a level of the vomer |
| 48 | Rt Choanae lat | Cho_r | Right posterior lateral border of the anterior nasopharyneal |
|  |  |  | space, posterior to the vomer |
| 49 | Lt Choanae Lat | Cho_l | Left posterior lateral border of the anterior nasopharyneal |
|  |  |  | space, posterior to the vomer |
| 50 | Mid Posterior Vomer-midVo | Vo | Point in the midline of the vomer level with the right and  left medial pterygoid plate |
|  |  | | |

### Table B**.** Formalised measurement definitions for label-based craniofacial biometry protocol with 3D T2w fetal MRI.

| **Anatomical group Measure Number** | **Measurement name** | **Abbreviation** | **Landmark points used** |
| --- | --- | --- | --- |
| 1 | Anterior skull base length | ASBL | Fc (1), PCP (11) |
| 1. Base of skull 2 | Posterior skull base length | PSBL | PCP (11), Ba (2) |
| 3 | Internal cranial Base angle (°) | CBA1 | Fc(1), PCP (11), Ba (2) |
| 4 | External cranial base angle (°) | CBA2 | PNS (6), H (4), Ba (2) |
| 2. Facial angles 5 | Fronto maxillary angle | FMA | Si (10), Fc (1), PCP (11) |
| 6 | Inferior facial angle | IFA | PCP (11), Fc (1), Lip (40), Chin (41) |
| 7 | Maxillary nasion mandibular angle | MNMA | ANS (3), Fc (1), Me (25) |
| 3. Oropharangeal 8 | Hard palate length | HPL | ANS (3), PNS (6) |
| 9 | Velopharangeal length | VPL | ANS (3), PNS (6), PPW (7) |
| 10 | Nasopharyngeal area (mm2) | NASO | PNS (6), H (4), Ba (2) |
| 11 | Oropharyngeal area (mm2) | ORO | PNS (6), Ba (2), TP (5) |
| 12 | Palatal width | PaW | Pa (Rt/Lt) (18,19) |
| 13 | Palatal height | PaH | Pav (20), RtPa, Lt Pa (18, 19) |
| 4. Nasal 14-15 | Choanae width (rt/Lt) | Cho_R/Cho_L | MPP (Rt/Lt) (46, 47), Vo (50) |
| 16 | Choanae height (mm) | ChoH | H (4), ANS (3), PNS (6) |
| 17 | Nasopharynx width | NPw | Cho (Lt/Rt) (48, 49) |
| 18 | Nasal bone | NB | NaIn (36), NB (12) |
| 19 | Prenasal thickness | PNTh | NaIn (36), NaO (37) |
| 20 | Occipital frontal diameter | OFD | Si (10), Oc (8) |
| 5. Cranial vault 21 | Bi parietal diameter | BPD | Pt (Rt/Lt) (15/16) |
| 22 | Head circumference | HC | SI (10), Oc (8), RtPt (15), LtPt (16) |
| 23 | Maximum cranial height | MCh | Ve (17), Ba (2) |
| 24-25 | Orbital distance | ROD/LOD | RO/LO_in (33/35), RO/LO_o (34/36) |
| 6. Orbits 26 | Interoccular distance | IOD | RO_in (33), LO_i (35) |
| 27 | Biocular distance | BOD | RO/LO_o (34/36) |
| 7. Maxilla 28 | Maxillary width | MxW | RtMx (21), LtMx (22) |
| 29 | Maxillary length | MxL | ANS (3), RtMx (21), LtMx (22) |
| 8. Mandible 30 | Mandibular Width | MdW | RtMd (23), LtMd (24) |
| 31 | Mandibular length | MdL | Me (25), RtMd (23), LtMd (24) |

### Table C**.** Formulae for automated biometry calculation based on 3D labels

| **Description** | **Formula** |
| --- | --- |
| The distance *d* between two labels  *P*_1_(*x*_1_*, y*_1_*, z*_1_) and *P*_2_(*x*_2_*, y*_2_*, z*_2_) | *d* = ✓(*x*_2_ − *x*_1_)2 +(*y*_2_ − *y*_1_)2 +(*z*_2_ − *z*_1_)2 |
| The angle *θ* between two lines  defined by labels *P*_1_(*x*_1_*, y*_1_*, z*_1_), *P*_2_(*x*_2_*, y*_2_*, z*_2_), and *Q*_1_(*x*_3_*, y*_3_*, z*_3_), *Q*_2_(*x*_4_*, y*_4_*, z*_4_) in 3D space | *θ* = arccos √ (*x*2−*x*1)(*x*4−*x*3)+(*y*2−*y*1)√(*y*4−*y*3)+(*z*2−*z*1)(*z*4−*z*3) ) (*x*2−*x*1)2+(*y*2−*y*1)2+(*z*2−*z*1)2 (*x*4−*x*3)2+(*y*4−*y*3)2+(*z*4−*z*3)2 |
| The distance *d* between a label  *P*(*x*_3_*, y*_3_*, z*_3_) and the center point of a line defined by labels  *Q*_1_(*x*_1_*, y*_1_*, z*_1_) and *Q*_2_(*x*_2_*, y*_2_*, z*_2_) | *d* = J(*x*1+*x*2−2*x*3)2+(*y*1+*y*2−2*y*3)2+(*z*1+*z*2−2*z*3)2  4 |
| The area *A* of a triangle from three  labels *P*_1_(*x*_1_*, y*_1_*, z*_1_), *P*_2_(*x*_2_*, y*_2_*, z*_2_), and *P*_3_(*x*_3_*, y*_3_*, z*_3_) in 3D space | *A* = ^1^ ∥**x**_1_(*x*_2_ − *x*_1_*, y*_2_ − *y*_1_*, z*_2_ − *z*_1_) × **x**_1_(*x*_3_ − *x*_1_*, y*_3_ − *y*_1_*, z*_3_ − *z*_1_)∥  2 |
| The length of an ellipse *L* using two  perpendicular lines (the OFD and BPD), each defined by labels  *P*_1_(*x*_1_*, y*_1_*, z*_1_), *P*_2_(*x*_2_*, y*_2_*, z*_2_), and  *Q*_1_(*x*_3_*, y*_3_*, z*_3_), *Q*_2_(*x*_4_*, y*_4_*, z*_4_) | *L* = 1*.*62 ×  (✓(*x*_2_ − *x*_1_)2 +(*y*_2_ − *y*_1_)2 +(*z*_2_ − *z*_1_)2 +✓(*x*_4_ − *x*_3_)2 +(*y*_4_ − *y*_3_)2 +(*z*_4_ − *z*_3_)2 |

### Table D. Demographics of the 10 cases selected for quantitative evaluation of the proposed biometry protocol and pipeline.

| ID | GA | Group | Sex | TE | Field Strength | HeadSVR quality |
| --- | --- | --- | --- | --- | --- | --- |
| 1 | 29.86 | T21 | Male | 180ms | 3.0T | Good |
| 2 | 35.43 | T21 | Male | 180ms | 3.0T | Moderate |
| 3 | 32.71 | T21 | Male | 250ms | 3.0T | Moderate |
| 4 | 33.71 | T21 | Female | 80ms | 1.5T | Good |
| 5 | 32.71 | T21 | Female | 80ms | 1.5T | Excellent |
| 6 | 31.86 | Control | Male | 80ms | 1.5T | Moderate |
| 7 | 32.86 | Control | Female | 80ms | 1.5T | Good |
| 8 | 29.43 | Control | Female | 80ms | 1.5T | Moderate |
| 9 | 30.00 | Control | Male | 180ms | 3.0T | Moderate |
| 10 | 35.14 | Control | Female | 250ms | 3.0T | Moderate |

**
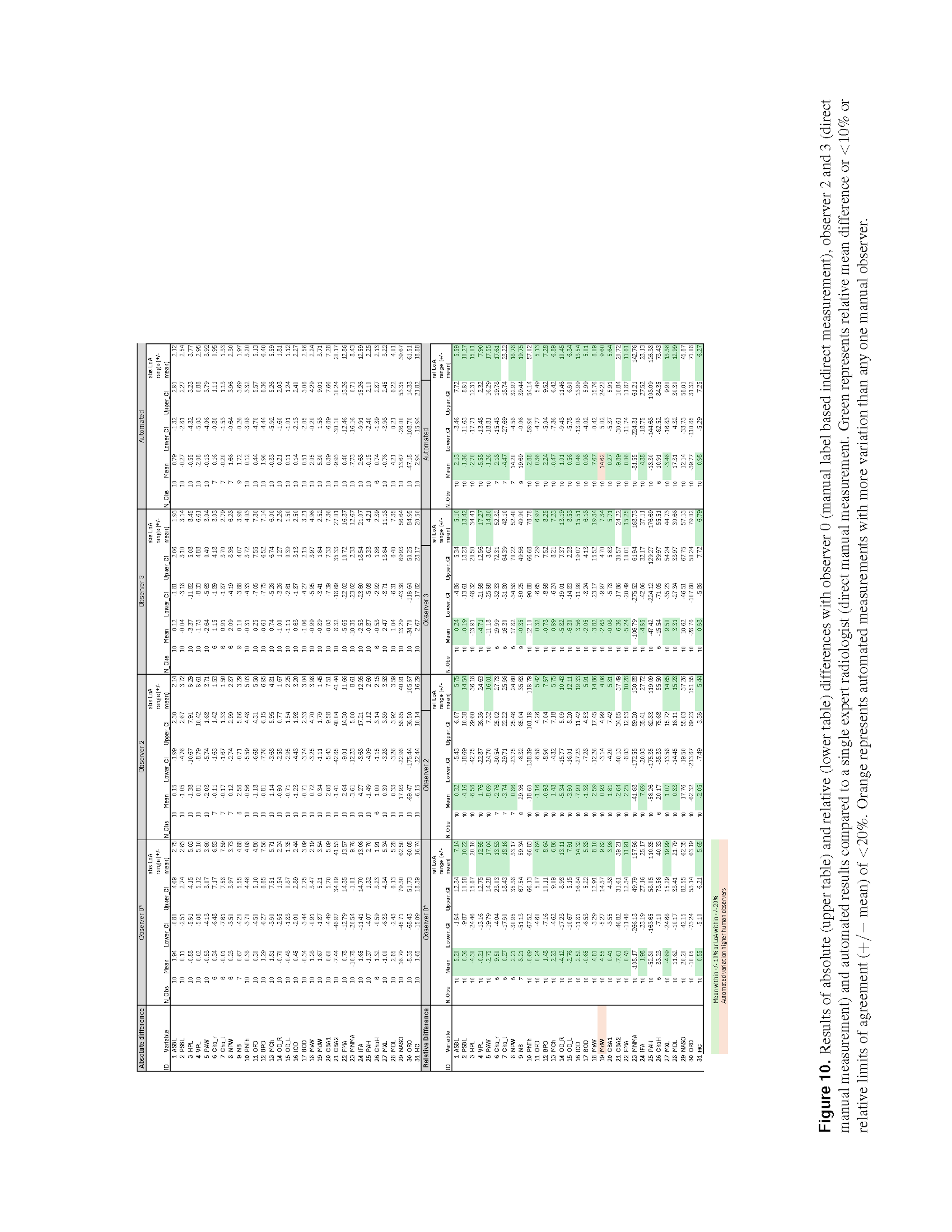
**

Table E. Results of absolute (upper table) and relative (lower table) differences with observer 0 (manual label based indirect measurement), observer 2 and 3 (direct manual measurement) and automated results compared to a single expert radiologist (direct manual measurement. Green represents relative mean difference or *<*10% or relative limits of agreement (+*/−* mean) of *<*20%. Orange represents automated measurements with more variation than any one manual observe


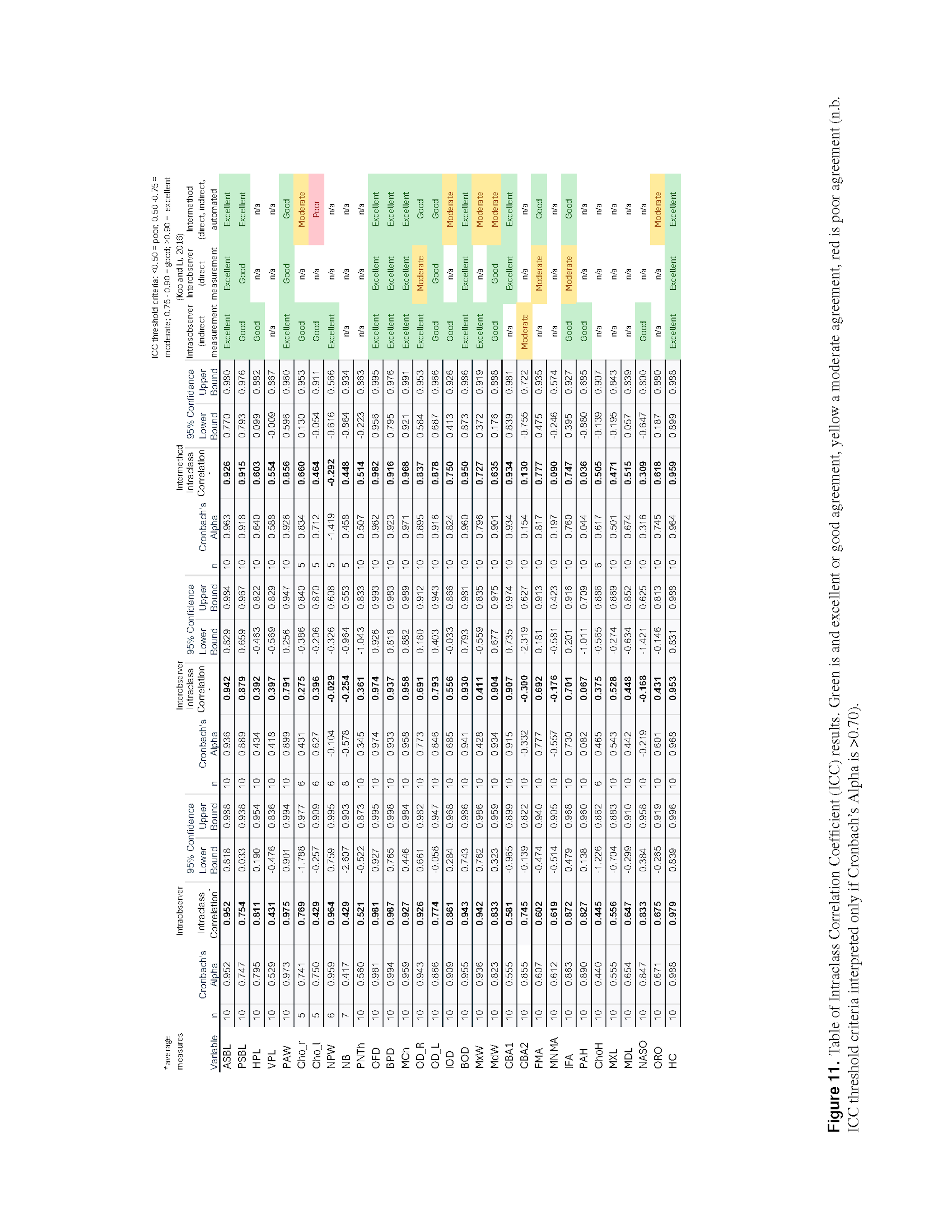


Table F. Table of Intraclass Correlation Coefficient (ICC) results. Green is and excellent or good agreement, yellow a moderate agreement, red is poor agreement (n.b. ICC threshold criteria interpreted only if Cronbach’s Alpha is >0.70).

### Table G. Table of number of subjects (n), mean measurement, and standard deviation (SD) - stratified by healthy control and T21 groups

|  |  | T21 (mean GA: 32.48) | | | Control (mean GA: 31.88) | | |
| --- | --- | --- | --- | --- | --- | --- | --- |
| Biometry ID | Biometry name, (unit) | n | Mean | SD | n | Mean | SD |
| 1 | ASBL, (mm) | 24 | 34.45 | 2.20 | 108 | 36.76 | 2.13 |
| 2 | PSBL, (mm) | 24 | 24.10 | 1.40 | 108 | 24.77 | 1.45 |
| 3 | HPL, (mm) | 24 | 24.69 | 2.10 | 108 | 26.02 | 1.85 |
| 4 | VPL, (mm) | 24 | 38.21 | 2.82 | 108 | 40.22 | 2.53 |
| 5 | PAW, (mm) | 24 | 24.76 | 1.71 | 108 | 24.88 | 1.72 |
| 6 | Cho_r, (mm) | 24 | 5.04 | 0.46 | 108 | 5.40 | 0.50 |
| 7 | Cho_l, (mm) | 24 | 5.68 | 0.45 | 108 | 5.94 | 0.53 |
| 8 | NPW, (mm) | 24 | 9.29 | 0.95 | 108 | 9.87 | 1.08 |
| 9 | NB, (mm) | 24 | 7.92 | 0.74 | 108 | 7.41 | 0.69 |
| 10 | PNTh, (mm) | 24 | 7.81 | 1.21 | 108 | 7.14 | 0.88 |
| 11 | OFD, (mm) | 24 | 97.82 | 5.41 | 108 | 101.05 | 6.34 |
| 12 | BPD, (mm) | 24 | 84.43 | 4.17 | 108 | 83.38 | 5.43 |
| 13 | MCh, (mm) | 24 | 79.80 | 4.13 | 108 | 80.36 | 4.26 |
| 14 | OD_R, (mm) | 24 | 17.18 | 0.88 | 108 | 17.35 | 0.98 |
| 15 | OD_L, (mm) | 24 | 17.53 | 0.99 | 108 | 17.80 | 1.07 |
| 16 | IOD, (mm) | 24 | 15.48 | 1.14 | 108 | 15.91 | 1.18 |
| 17 | BOD, (mm) | 24 | 49.95 | 2.64 | 108 | 50.64 | 2.77 |
| 18 | MxW, (mm) | 24 | 23.90 | 1.62 | 108 | 24.25 | 1.67 |
| 19 | MdW, (mm) | 24 | 30.42 | 1.86 | 108 | 30.89 | 2.26 |
| 20 | CBA1, ( ^0^ ) | 24 | 131.20 | 3.76 | 108 | 129.27 | 4.31 |
| 21 | CBA2, ( ^0^ ) | 24 | 116.89 | 5.72 | 108 | 115.39 | 7.50 |
| 22 | FMA, ( ^0^ ) | 24 | 116.28 | 4.56 | 108 | 114.04 | 4.99 |
| 23 | MNMA, ( ^0^ ) | 24 | 17.66 | 2.19 | 108 | 17.91 | 2.27 |
| 24 | IFA, ( ^0^ ) | 24 | 55.16 | 5.22 | 108 | 48.97 | 4.95 |
| 25 | PAH, (mm) | 24 | 3.28 | 0.63 | 108 | 3.00 | 0.58 |
| 26 | ChoH, (mm) | 24 | 3.30 | 0.54 | 108 | 3.22 | 0.59 |
| 27 | MXL, (mm) | 24 | 24.99 | 1.95 | 108 | 26.23 | 1.80 |
| 28 | MDL, (mm) | 24 | 19.02 | 1.68 | 108 | 19.27 | 1.53 |
| 29 | NASO, (*mm*^3^) | 24 | 58.42 | 7.18 | 108 | 63.88 | 9.37 |
| 30 | ORO, (*mm*^3^) | 24 | 181.41 | 32.81 | 108 | 173.97 | 33.06 |
| 31 | HC, (mm) | 24 | 289.62 | 14.46 | 108 | 296.01 | 18.20 |

### Table H. Summary of included datasets for the 3rd trimester T21 and age-matched control cohorts, by GA, MRI protocol, fetal sex and ethnicity

|  |  | **T21** | **Control** | **Total count all,**  **n** |
| --- | --- | --- | --- | --- |
| **GA** | Range  Mean (SD) | 29.86 - 35.71  32.48 (1.85) | 29.00 - 36.00  31.88 (2.01) | -  - |
| **MRI Protocol, n (%)** | 1.5T / 80ms  3T / 180ms  3T / 250ms | 4 (16.6)  17 (70.8)  3 (0.1) | 34 (31.5)  25 (23.1)  49 (45.4) | 38  42  52 |
|  | **MRI protocol total, n**  **(%)** | **24(100)** | **108 (100)** | 132 |
| **Fetal Sex, n (%)** | Female  Male Unknown | 10 (41.7)  10 (41.7)  4 (5.4) | 53 (49.1)  54 (50.0)  1 (0.9) | 63  64  5 |
|  | **Fetal sex total,n (%)** | **24 (100)** | **108 (100)** | 132 |
| **Ethnicity, n (%)** | Asian  Black Other Unknown White | 1 (4.2)  1 (4.2)  0 (0)  21 (87.5)  1 (4.2) | 10 (9.3)  4 (3.7)  3 (2.8)  5 (4.6)  86 (79.6) | 11  5  3  26  87 |
|  | **Ethnicity total, n** (%) | **24 (100)** | **108 (100)** | 132 |

| **Variable** | **Bestfit line equation** | **SD equation** | **Mean bestfit method** |
| --- | --- | --- | --- |
| ASBL | 0.7191 *GA + 13.340 | 0.039 *GA + 0.221 | Linear |
| PSBL | 28.312 + -0.6771 * GA + 0.018 *GA*GA | 0.060 * GA + -0.898 | Quadratic |
| HPL | 0.6207 * GA + 6.230 | 0.068 * GA + -0.843 | Linear |
| VPL | 58.7882 + -2.0538 * GA + 0.0460 * GA*GA | -0.031 * GA + 2.508 | Quadratic |
| PAW | 0.6259 * GA + 4.925 | 0.042 * GA + -0.178 | Linear |
| Cho_r | 0.1199 * GA + 1.575 | 0.019* GA + -0.173 | Linear |
| Cho_l | 0.1438 * GA + 1.359 | 0.049 * GA + -1.150 | Linear |
| NPW | 0.1517 * GA + 5.0364 | -0.022 * GA + 1.71 | Linear |
| NB | 35.307 + -1.940 * GA + 0.032 * GA*GA | 0.008 * GA + 0.188 | Quadratic |
| PNTh | 0.211 * GA + -0.926 | -0.013* GA + 1.085 | Linear |
| OFD | 113.735 + -2.971 * GA + 0.0803 * GA*GA | 0.118 * GA + 0.432 | Quadratic |
| BPD | 1.885 * GA + 23.520 | 0.037 * GA + 2.485 | Linear |
| MCh | 1.410 * GA + 34.461 | 0.119 * GA + -1.101 | Linear |
| OD_R | 0.3607 * GA + 5.856 | 0.011 * GA + 0.242 | Linear |
| OD_L | 0.393 * GA + 5.281 | 0.037 * GA + -0.501 | Linear |
| IOD | 0.2642 * GA + 7.485 | 0.023 * GA + 0.255 | Linear |
| BOD | 1.049 * GA + 17.203 | 0.037 * GA + 0.435 | Linear |
| MxW | 0.609 * GA + 4.849 | 0.081 * GA + -1.470 | Linear |
| MdW | -19.0189+ 2.345 * GA + -0.024 * GA*GA | 0.002 * GA + 1.572 | Quadratic |
| CBA1 | 0.230 * GA + 120.529 | 0.266 * GA + -4.4712 | Linear |
| CBA2 | 1.301 * GA + 79.057 | 0.266 * GA + -4.471 | Linear |
| FMA | 0.091 * GA + 109.872 | 0.140* GA + 0.693 | Linear |
| MNMA | 0.159 * GA + 13.787 | 0.0158 * GA + 1.795 | Linear |
| IFA | 0.253 * GA + 42.129 | 0.206 * GA + -1.307 | Linear |
| PAH | 0.067 * GA + 0.858 | 0.004 * GA + 0.392 | Linear |
| ChoH | 0.0129 * GA + 2.880 | 0.023* GA + -0.126 | Linear |
| MXL | 55.528 + -2.427 * GA + 0.047 * GA*GA | 0.005 * GA + 1.058 | Quadratic |
| MDL | 33.245 + -1.336* GA + 0.028 * GA*GA | 0.0321 * GA + 0.136 | Quadratic |
| NASO | 2.376 * GA + -2.227 | 0.0142 * GA + 8.092 | Linear |
| ORO | 9.157 * GA + -117.920 | 1.535 * GA + -21.413 | Linear |
| HC | 322.684 + -7.907 * GA + 0.221 * GA*GA | -0.145 * GA + 16.694 | Quadratic |

### Table I. Best linefit and Standard Deviation (SD) regression equations and method, for each biometric growth chart. GA = GA. Note: all SD regression formulae have linear bestfit method.

| **Dependent Variable** | **Test parameter**  **with covariate** | **B** | **Robust**  **Std. Error** | **t** | **Sig.** | **95% C.I.**  **Lower Bound** | **95% C.I.**  **Upper Bound** | **Partial**  **Eta Squared** | **Noncent.**  **Parame- ter** | **Observed**  **Power** |
| --- | --- | --- | --- | --- | --- | --- | --- | --- | --- | --- |
| Anterior Skull Base Length,  ASBL | T21 with GA* | -  31.631 | 6.334 | -  4.994 | 0.000 | -44.163 | -19.099 | 0.162 | 4.994 | 0.999 |
| Hard Palate Length, HPL | T21 with GA* | -1.682 | 0.329 | -  5.107 | 0.000 | -2.334 | -1.030 | 0.168 | 5.107 | 0.999 |
| Velopharyngeal Length, VPL | T21 with GA* | -2.536 | 0.438 | -  5.784 | 0.000 | -3.403 | -1.668 | 0.206 | 5.784 | 1.000 |
| Occipitofrontal Diameter, OFD | T21 with GA* | -4.389 | 0.908 | -  4.835 | 0.000 | -6.185 | -2.593 | 0.153 | 4.835 | 0.998 |
| Biparietal Diameter, BPD | T21 with GA§ | 0.297 | 0.677 | 0.438 | 0.662§ | -1.043 | 1.636 | 0.001 | 0.438 | 0.072 |
| Inferior Facial Angle, IFA | T21 with GA* | 7.042 | 1.563 | 4.505 | 0.000 | 3.949 | 10.135 | 0.136 | 4.505 | 0.994 |
| Maxillary Length, MXL | T21 with GA* | -1.608 | 0.308 | -  5.217 | 0.000 | -2.217 | -0.998 | 0.174 | 5.217 | 0.999 |
| Nasopharyngeal Area, NASO | T21 with GA* | -8.483 | 1.777 | -  4.774 | 0.000 | -11.999 | -4.968 | 0.150 | 4.774 | 0.997 |
| * = Large effect size  § = non-significant result and at risk of Type II error GA = Gestational Age | | | | | | | | | | |

Table J. ANOVA results for statistically significant biometry with largest effect size in T21 cohort (ABSL, HPL, VPL, OFD, IFA, NASO) and BPD as a non-significant results. Results corrected with robust standard errors.
